# Towards the elimination of visceral leishmaniasis as a public health problem in east Africa: reflections on an enhanced control strategy and a call for action

**DOI:** 10.1016/S2214-109X(21)00392-2

**Published:** 2021-11-16

**Authors:** Jorge Alvar, Margriet den Boer, Daniel Argaw Dagne

**Affiliations:** a Drugs for Neglected Diseases initiative, Geneva, Switzerland; b Centro Nacional de Medicina Tropical, Instituto de Salud Carlos III, Madrid, Spain; c Médecins Sans Frontières, London, UK; d Department of Control of Neglected Tropical Diseases, WHO, Geneva, Switzerland

## Abstract

East Africa is the world region most affected by visceral leishmaniasis, accounting for 45% of cases globally that were reported to WHO in 2018, with an annual incidence that is only slightly decreasing. Unlike southeast Asia, east Africa does not have a regional approach to achieving elimination of visceral leishmaniasis as a public health problem. The goal of the WHO 2021–30 Neglected Tropical Diseases road map is to reduce mortality caused by the disease to less than 1%. To achieve this goal in east Africa, it will be necessary to roll out diagnosis and treatment at the primary health-care level and implement evidence-based personal protection methods and measures to reduce human–vector contact. Investment and collaboration to develop the necessary tools are scarce. In this Health Policy paper, we propose a strategic framework for a coordinated regional approach in east Africa for the elimination of visceral leishmaniasis as a public health problem.

## Introduction

Visceral leishmaniasis (also known as kala-azar) is a vector-borne systemic disease that is fatal if left untreated. It is listed by WHO as a neglected tropical disease. Worldwide, the incidence of visceral leishmaniasis has reduced by more than 30% since 2016.^1^ WHO's 2021–30 neglected tropical diseases road map has set an ambitious target for global elimination of the disease. Milestones are less than 1% mortality due to primary visceral leishmaniasis in 56 countries by 2025, and in 64 countries by 2030 (which is 85% of the total number of endemic countries).^2^ East Africa is currently the most affected region in the world, accounting for 45% of visceral leishmaniasis cases reported to WHO globally in 2018.^1^ Here, visceral leishmaniasis remains a neglected and under-reported disease and has catastrophically impoverishing effects on some of the most vulnerable populations.^3^, ^4^ Visceral leishmaniasis is endemic in 78 countries but mainly affects economically disadvantaged populations in east Africa, southeast Asia, and Brazil.^1^ Historically, the highest burden of visceral leishmaniasis was found in southeast Asia, with an estimated 270 900 cases in 2007.^5^ However, since the Ministries of Health from Bangladesh, India, and Nepal signed a memorandum of understanding in 2005 to achieve elimination as a public health problem, the caseload has decreased steadily.^6^ Elimination thresholds were reached in Bangladesh in 2013, and in Nepal in 2016; in 2019, only 3128 cases were reported in these two countries combined. National political commitment, effective tools to diagnose and treat the disease (including early case detection), and collaboration with partners were the key drivers to the success of the elimination programme and, if continued, should prevent a re-emergence of the disease. In this paper, we propose a coordinated regional approach for attaining the control and elimination of visceral leishmaniasis in east Africa.

## East Africa: the context

### Epidemiology

In east Africa, visceral leishmaniasis is caused by the parasite *Leishmania donovani*. Endemic foci are found in remote areas, underserved by health care, in Ethiopia, Eritrea, Kenya, Somalia, South Sudan, Sudan, and Uganda. The caseload in the region varies from country to country, and it has not decreased substantially over the past 5 years (appendix p 2). Due to economically driven migration or massive population displacements during conflicts, endemic areas are continually expanding. Immunologically naive populations entering endemic areas are at high risk of infection, while those migrating from endemic areas can introduce visceral leishmaniasis to areas where the vector is present but the disease is not yet endemic.^7^ In both scenarios, large-scale outbreaks have frequently occurred, especially in Ethiopia, Kenya, and South Sudan.^7^, ^8^ Outbreaks are often detected at a late stage due to poor surveillance or because they are initially mistaken for malaria or other acute febrile illnesses, since visceral leishmaniasis is mostly unknown outside endemic areas. If not addressed in a timely manner, these outbreaks can cause very high mortality.^7^, ^8^ During outbreaks, individuals of all ages, and men and women, are roughly equally affected whereas, in longstanding endemic foci, 60% of the cases occur in children younger than 15 years;^9^ however, in Ethiopia, seasonal migrant labourers travelling to endemic zones for the planting and harvesting of cash crops are at highest risk. This group also has the highest rate (approximately 20%) of HIV and visceral leishmaniasis co-infection worldwide.^5^ HIV and other comorbidities or co-infections are substantial risk factors for developing clinical visceral leishmaniasis, as is malnutrition.

### Access to care

In the absence of evidence-based, effective, and scalable vector control strategies, national control programmes in east Africa have had little choice but to rely on early diagnosis and treatment to curb transmission and reduce mortality. Despite an aspiration to universal health coverage, in remote endemic areas, health systems are often poorly developed and under-resourced, and they have little capacity to integrate the complex diagnostic and treatment services for visceral leishmaniasis within an already limited package of basic health services. Diagnosis and treatment are only provided in a designated number of government hospitals or advanced health centres in endemic areas. In the past 15 years, some progress has been made in parallel with improving health systems and donor support, and the number of health facilities providing diagnosis and treatment has increased in some countries. However, understaffing and interrupted supply of diagnostics and drugs remain common.^3^, ^4^ Access to these health facilities is often hindered by extreme poverty and the large distances from the remote locations where patients live and work. Although diagnosis and treatment are provided free of charge, the combined out-of-pocket costs of a disease episode, including transport, loss of wages, and cost of hospital stays, often lead to financial catastrophe.^3^, ^4^ In Somalia and South Sudan, insecurity, due to conflict, impedes the provision of visceral leishmaniasis treatment, and the problem of poor access is compounded by the absence of a functional socioeconomic infrastructure.^10^

### Surveillance

A strong surveillance system is crucial for the early detection of visceral leishmaniasis outbreaks and for the monitoring of disease trends. In east Africa, visceral leishmaniasis surveillance differs from country to country due to variations in health infrastructure, the particular epidemiology of the disease, the status of the national health system, and the way the national health information system has been set up. The development and use of recording and reporting forms with standard indicators and advances in software tools, coupled with intensive capacity building activities by WHO and partners, have resulted in improved surveillance. Although the overall reporting rate from the WHO African region is low at 43%, east African countries have instituted improved surveillance with enhanced timeliness and completeness for visceral leishmaniasis.^1^

### Case management

Visceral leishmaniasis in east Africa is diagnosed by a rK39-antigen-based rapid test, used in clinically suspected cases. Because this test has a suboptimal sensitivity of around 85%,^11^ the following additional diagnostic tests are often necessary: microscopy detection of amastigotes in stained smears from lymph node punctures, bone marrow or spleen aspirates, and the direct agglutination test. Another drawback is that the rapid diagnostic test cannot be used for verifying cure, and it is less specific if typical symptoms are absent. The first-line treatment consists of a 17-day combination regimen of antimonials (namely sodium stibogluconate) and paromomycin which, due to its potential toxicity, is not suitable for all patients. Patients who are pregnant, severely ill, or older than 45 years are therefore treated with multiple-dose liposomal amphotericin B and HIV co-infected patients with a liposomal amphotericin B-miltefosine combin-ation instead.^12^ If treated in a timely manner, cure rates are generally high, although HIV and TB co-infections cause high relapse and mortality rates.

Post kala-azar dermal leishmaniasis is a skin sequela of visceral leishmaniasis that manifests usually within 6 months of treatment in a variable proportion of cured patients; in Sudan, prevalence is estimated to be up to 20%, in other countries it is not well documented. This condition is often mild and self-limiting, and most patients do not report to clinics.^13^ In severe cases, a treatment of 60–90 days of antimonials is necessary, which is highly toxic and demanding to patients and to hospitals.

A major drawback of the currently used diagnostic algorithms and treatments is that they are not suited for roll-out beyond the hospital or well equipped health centre. New treatments are under development: a clinical trial of a 14-day treatment with paromomycin and miltefosine is underway, with results expected in 2021, and there are new compounds that show promise as oral treatments of 10-day duration or less.^14^ A safe oral drug might offer prospects for chemoprophylaxis, to be given during the transmission season. Therapeutic vaccines could be an option for preventing relapse and post kala-azar dermal leishmaniasis. The development of a more sensitive rapid test, preferably one that detects antigens, should be a priority. A simple, low cost DNA-detecting CRISPR-Cas-based rapid test is a promising option to be explored.^15^

### Transmission and vector control

*Phlebotomus orientalis* (a species of sand fly) are responsible for the transmission of *Leishmania donovani* in the northern endemic foci; Sudan, north Sudan, and north Ethiopia. These flies are abundant in savannahs dominated by balanites and acacia trees, and they breed inside the cracks of black cotton soil. The flies rarely go indoors, but they bite humans in courtyards and other peridomestic habitats. They show marked seasonality, with a peak abundance in the dry season (March–June).^16^ *Phlebotomus martini* (another sand fly species) are the principal vector in the southern foci, which includes Somalia, Kenya, Uganda, and south Ethiopia, and breed inside termite mounds. Sleeping or working outdoors during the hot, dry season in proximity of balanites or acacia trees (or, in the southern foci, near termite mounds) increases the risk of infection.

Patients with visceral leishmaniasis or post kala-azar dermal leishmaniasis act as infectious reservoirs of visceral leishmaniasis and, due to their high parasite loads, patients co-infected with HIV and visceral leishmaniasis can be considered superspreaders.^17^ Wild mammals, found only in specific ecosystems, form a zoonotic reservoir and might also contribute to transmission.

Control measures for the sand fly vectors of *L donovani* in east Africa have rarely been studied, and they are not implemented by national control programmes. Although there is evidence that insecticide-impregnated bednets reduce exposure to the bites of *P orientalis*,^18^ these are not appropriate for most settings due to human behavioural factors such as sleeping outside during the dry and hot transmission season.

## A prospective approach: towards elimination of visceral leishmaniasis in east Africa

Visceral leishmaniasis control is challenging in east Africa; more so than in southeast Asia. The reasons include: the absence of a highly sensitive rapid test for the diagnosis of primary visceral leishmaniasis; a complex treatment regimen that can only be implemented at a well equipped health centre or hospital; the absence of evidence-based personal protection measures and measures to reduce the exophilic vector density; and the fact that at-risk populations living in poverty reside in areas that are insecure due to conflict, very remote from hospitals, and often inaccessible to health-care workers. Patients often suffer from malnutrition, co-infections, and comorbidities. Additionally, only when further progress is made towards universal health coverage can elimination of visceral leishmaniasis be achieved in the endemic regions of east Africa, where general health system strengthening efforts are needed. These factors have discouraged policy makers and the international donor community from designing and investing in programmes to eliminate visceral leishmaniasis as a public health problem and to reduce its toll on human lives. Consequently, national control programmes are poorly funded, deprioritised, and have relied on external support from WHO, the UK Foreign, Commonwealth and Development Office, Médecins Sans Frontières, the Drugs for Neglected Diseases initiative, and other non-governmental organisations and donors. There is no regional strategy or resolution by WHO regional committees or other regional bodies, and there is little political willingness to commit dedicated funding to visceral leishmaniasis control.

In southeast Asia, the regional visceral leishmaniasis elimination programme benefited from strong political commitment. Bangladesh, India, and Nepal signed a renewable memorandum of understanding, while the programme was fully endorsed and supported by WHO and stakeholders. WHO's Regional Office for South-East Asia instituted a 5-year rolling strategic framework and a high-level regional technical advisory group that reports to the regional director. This approach, in combination with country leadership and governments' allocation of dedicated funds, the donation of the first-line treatment (liposomal amphotericin B used as first line treatment in Asia) by Gilead Sciences (Foster City, CA, USA), and various long-term donor agreements eventually led to a highly successful programme that has reduced the incidence of visceral leishmaniasis to near-elimination levels.^19^

We propose that a similar coordinated regional approach in east Africa for the elimination of visceral leishmaniasis as a public health problem is the best way forward to address the ongoing neglect. We outline a stepwise approach, where the initial investment in operational research is of paramount importance. Table 1 shows our proposal for a strategic framework. At the centre of the strategy lies improved case detection via decentralised diagnosis and treatment, which is key to reducing the time between first symptoms and treatment. This approach could also contribute to improving access to diagnosis and treatment and reducing the number of undetected cases. Decentralised diagnosis and treatment will require a highly sensitive and specific rapid test and short-course, safe and, ideally, oral treatments for both visceral leishmaniasis and post kala-azar dermal leishmaniasis. The development and introduction of both a new rapid test and short-course treatments need to be encouraged and fast-tracked (table 2). Integrated interventions in diagnosis with other disease groups can also be explored, such as the development of a standardised approach to the diagnosis of persistent fevers at the primary health-care level.^23^

**Table 1:** Framework for a strategy for preparedness towards visceral leishmaniasis elimination in east Africa

	**Objectives**	**Activities**	**Indicators**	**Progress needed and challenges**
Case management	Early case detection; improve case management; reduce mortality; prevent stock-out of diagnostics and drugs	Timely forecast and supply of diagnostics and drugs; service decentralisation; development of referral capacity; regular training of health workers; free care for visceral leishmaniasis and comorbidities; screening patients with HIV for visceral leishmaniasis	Number of stock-outs; time from onset to treatment; financial burden to patients; compliance to treatment for visceral leishmaniasis, HIV-visceral leishmaniasis, and PKDL treatment; geographical access to diagnosis and treatment; incidence and mortality rate	Better rapid diagnostic test for visceral leishmaniasis; better (oral) drugs for treatment and prophylaxis of visceral leishmaniasis and PKDL; therapeutic vaccines; better PKDL diagnosis; improved and new visceral leishmaniasis and PKDL test of cure; telemedicine for PKDL diagnosis
Integrated vector management	Reduce contact with sandflies; monitor insecticide efficacy and resistance; establish entomology network	Mapping environmental risks; scaling up integrated vector management in different settings; training personnel in vector management; developing regional stocks of insecticide and integrated vector management tools; building entomology research centres and insectaries	Percentage covered by prevention measures; regional stocks of insecticide and integrated vector management tools; patients with visceral leishmaniasis, PKDL, or co-infection with HIV use vector control tools; the presence of a functional entomology network; the presence of a functional vector surveillance system	Vector control operational guidelines and tools; entomology experts and careers; regional centres of excellence
Effective surveillance	Map areas by risk level; map visceral leishmaniasis health services; improve epidemiological data on visceral leishmaniasis, case fatality rate, PKDL, and HIV-visceral leishmaniasis; outbreak preparedness and response	Implementing tools for data collection and analysis; outbreak preparedness and response; capacity building in surveillance; identifying the most affected groups	Tools routinely in use; timely response to outbreaks; sufficient personnel trained; case detection in new and existing endemic areas; presence of a functional surveillance system; whether visceral leishmaniasis is a notifiable disease	Tools are partially used; robust information system needed; outbreak response is led by non-governmental organisations; visceral leishmaniasis is not a notifiable disease
Social mobilisation	Increase awareness and establish behavioural change in health seeking behaviour and use of prevention tools	Information, education, and communication and behavioural change communication activities; targeted advocacy for the most affected groups; periodic knowledge, attitude, and practice studies	Most affected groups use integrated vector management tools; local activists are in place; most affected groups seek diagnosis and treatment promptly	Multilingual and cultural approach; migrant and displaced populations difficult to reach; most disease occurs in areas that are remote and difficult to access; poor socio-economic and health infrastructure
Operational research	Improve visceral leishmaniasis (in the presence and absence of HIV) and PKDL diagnosis and treatment; improve access to diagnosis and treatment; develop innovative vector control tools and test ivermectin for vector control	Studies of access barriers in the most affected groups; studies to improve diagnosis and treatment for visceral leishmaniasis, PKDL, and HIV-visceral leishmaniasis; studies on sandflies bionomics and vector control	Availability of new diagnosis and treatment; availability of new vector control tools; better knowledge of sand fly behaviour, and vector control strategy is planned accordingly; access issues known and strategies developed	Fragile security situation; limited expertise and dependence on external technical and financial support; unknown role of PKDL, asymptomatic carriers, and animal reservoir in transmission

The overall impact of this strategy would include: new and improved tools for diagnosis and treatment for visceral leishmaniasis and PKDL; incidence and fatality rate reduced and large outbreaks prevented; catastrophic expenses reduced to negligible level; population protected from vector transmission; and fast access to diagnosis and treatment for most affected groups enabled. PKDL=post kala-azar dermal leishmaniasis.

**Table 2:** Key differences in visceral leishmaniasis epidemiology, policy strategies, and control in east Africa and southeast Asia, and unmet needs in operational research in east Africa, by strategy phase

		**East Africa**	**Southeast Asia**	**Operational research needed in east Africa**	**Relevant phase**
Major endemic countries		Ethiopia, Kenya, Somalia, South Sudan, Sudan, Eritrea, and Uganda	Bangladesh, India, and Nepal	..	NA
Parasite		*Leishmania donovani*	*L donovani*	..	NA
Vectors					
	Main vectors	*Phlebotomus orientalis, Phlebotomus martini,* and *Phlebotomus celiae*	*Phlebotomus argentipes*	Bionomy studies of *P orientalis, P martini,* and *P celiae*; innovative tools to control outdoor and peridomestic vector; individual protection methods for seasonal agricultural workers and pastoralists; mass drug administration of ivermectin during transmission season for insecticidal effect; acceptability of different control methods	Preparatory
	Vector behaviour	Exophilic; sylvatic and peridomestic	Endophilic; predominantly indoors but also peridomestic	Insecticide resistance and rotation studies; role of asymptomatic carriers and patients with PKDL in transmission of infection to the vector	Consolidation
	Vector control	No proven, effective, or scalable vector control strategy; integrated vector management is recommended	Indoor residual spraying is effective; integrated vector management is recommended	Role of the animal reservoir in transmission	Preparatory
Diagnosis					
	Diagnostic algorithm	Diagnostic algorithm includes clinical assesment followed by a rapid diagnostic test, a direct agglutination test, and parasite detection via microscopy	Clinical and rapid diagnostic tests used; parasite detection via microscopy only used for relapses	..	NA
	Performance of rapid diagnostic tests	Simple point-of-care rK39-antigen-based rapid diagnostic test sensitivity (85%) is lower than that in southeast Asia, but specificity is similar;^11^ direct agglutination test has a higher sensitivity (93%) and specificity (96%) than the rapid diagnostic test;^20^ diagnosis of primary infection is complex and needs well equipped laboratories	Simple point-of-care rK39-antigen-based rapid diagnostic tests have high sensitivity (97%) and specificity (90%)^11^ and can be used for the diagnosis of all primary infections at peripheral health-care facilities; direct agglutination tests have not been deployed widely because the rapid diagnostic tests perform much better	Improved rapid diagnostic tests with high sensitivity and specificity to decentralise diagnosis	Preparatory
	Delay to treatment	Delay from first symptoms to treatment of 1–2 months^21^	Delay from first symptoms to treatment of 1–3 months^22^	Non-invasive test of cure for visceral leishmaniasis and PKDL; non-invasive diagnosis of PKDL; support with telemedicine	Attack
Treatment and prevention					
	Clinical severity	Severe disease; co-infections and comorbidities, and associated malnutrition are common; often needs management in hospital	Moderate disease; can generally be managed at primary health-care level	Biomarkers of evolution of visceral leishmaniasis towards PKDL; oral short course treatments for therapeutic or prophylactic purposes (mass drug administration during transmission season); therapeutic vaccines (visceral leishmaniasis and PKDL); monitoring drug resistance	Consolidation
	Treatment	Suboptimal efficacy, administration of multiple injections, long duration, and serious side-effects; sodium stibogluconate plus paromomycin combination as the first-line option has an efficacy rate of 91·4%; liposomal amphotericin B has variable efficacy; treatment is complex and given at a designated hospital or well equipped health centre; clinical mentoring teams ensure standard of care	Highly effective and safe single-dose regimen; single-dose liposomal amphotericin B is the first-line option, with an efficacy of >97%; relapses are treated with multiple doses of liposomal amphotericin B or combinations of miltefosine plus paromomycin; treatment is given at designated primary health-care centres	Establishment of a network of referral centres; monitoring drug efficacy; better understanding of reasons for delay in access to diagnosis and treatment	Preparatory
Epidemiology					
	Epidemiological trends and interventions since 2005	No significant decline in incident cases; insufficient tools; recurrent epidemics, especially in Kenya, South Sudan, and Ethiopia; some reduction in mortality rate; high *Leishmania* and HIV co-infection rate, although there is a declining trend with decreasing HIV incidence globally	Declining incidence: elimination target achieved in Bangladesh and Nepal pending WHO validation; significant reduction in mortality rate; HIV and *Leishmania* co-infection rate is very low especially in Bangladesh and Nepal	Validation of the historical foci and estimate the population at risk, incidence, and mortality; environmental risk maps to predict outbreaks; viability of mobile clinics for mobile and displaced populations; pharmacovigilance through sentinel sites; to determine the elimination threshold required for resurgence of cases	Preparatory
	Most affected groups	Children, low-income households in rural villages, seasonal agricultural workers (in Ethiopia), nomadic pastoralists, and displaced populations	Children and adults, particularly those in low social castes (Musharat)	..	NA
	Transmission dynamics and ecology	Predominantly anthroponotic with zoonotic aspects; lowlands, acacia trees, black cotton soil, and termite hills	Anthroponotic; poor housing conditions and waste management, open sewerage, and cattle sheds	..	NA
	Endemic area	Vast, scattered, and remote; long distances to treatment centres; poor socio-economic infrastructure and health systems	Densely populated areas with good access to primary health-care centres	..	NA
	Programme target or goal	Control, with no defined target threshold	Elimination as a public health problem, defined as <1 case per 10 000 population at implementation-unit level	..	NA
	Regional political commitments	No agreements among endemic countries; no WHO regional resolution or strategic action framework; control programmes rely on external support (from WHO, Médecins Sans Frontières, Drugs for Neglected Diseases initiative, the UK's Foreign, Commonwealth, and Development Office, etc), WHO-led coordination of national programmes and partners (through periodic review meetings) and Drugs for Neglected Diseases initiative-led leishmaniasis east Africa platform (LEAP) for clinical trials on drugs; no long-term donor commitments	Strong political commitment forged; a signed renewable memorandum of understanding among the endemic countries with WHO regional committee resolution; a 5-year rolling strategic framework (WHO) and a high level Regional Technical Advisory Group reporting to the Regional Director; donation programme for first-line treatment	..	NA
	Programme strategy for access to care	Passive case detection; diagnosis and treatment provided for free; largely no support for bed occupancy fees and provision of meals, comorbidities, or transport; active case detection only in the event of outbreaks	Active case detection as a programme strategy; diagnosis and treatment provided for free, plus cash transfers for patients completing treatment as compensation for wage losses	..	NA

PKDL=post kala-azar dermal leishmaniasis. NA=not applicable.

In this strategy, the diagnosis and treatment of visceral leishmaniasis and post kala-azar dermal leishmaniasis should be provided free of charge at the primary health-care level; and a referral system including free transport, waiving hospital bed fees, provision of meals, and management of malnutrition and concomitant infections needs to be provided for complex cases. Regular training and mentoring of health workers should be in place, using innovative approaches, which can include telemedicine (mainly for post kala-azar dermal leishmaniasis), a visceral leishmaniasis person as a point of contact, or a helpline. The detection of patients who are co-infected with HIV could be improved by screening patients with HIV for visceral leishmaniasis in endemic regions. Because these patients are prone to relapse and are infectious reservoirs, they should be regularly monitored. Post kala-azar dermal leishmaniasis is also a proven infectious reservoir, and our strategy to reduce the case load includes finding and treating these patients. Detection among leprosy-negative skin lesion cases from the hospitals treating leprosy can be an effective integrated approach. Because visceral leishmaniasis in east Africa is likely to occur as outbreaks, particularly during mass displacement within areas where the vector is present, surveillance and the provision of prevention, diagnosis, and treatment should be ensured.

Since evidence-based, innovative approaches to control human–vector interactions in visceral leishmaniasis are absent for the east African region, in our strategy, we recommend the following interventions should be implemented immediately, if proven successful after evaluation (table 2). First, personal protection, via topical insect repellents applied to the skin, is a key intervention. These repellents are not traditionally used in visceral leishmaniasis, and they are unaffordable for the most affected populations, but they are proven to repel sandflies.^24^ This intervention is most promising for the large group of seasonal migrant workers in Ethiopia who are exposed to these vectors of visceral leishmaniasis and to malaria-carrying mosquitos during night-time work activities. Second, outdoor fence spraying with pyrethroids is a promising intervention. A pilot study showed this method was effective in reducing the peridomestic sand fly density in individual compounds in Sudanese communities.^25^ Finally, the potential role of mass drug administration for humans and livestock with ivermectin during the vector biting season as a complementary measure, as is currently evaluated for malaria vector control, is an option to be explored.^26^

It is feasible to develop and implement a road map for the control and elimination of visceral leishmaniasis during the next decade in east Africa. A regional technical advisory group under the leadership of WHO should be established, as was done in southeast Asia. Its initial task would be to analyse and define the appropriate milestones and elimination thresholds for the region, and to develop a strategic action framework. In parallel, political and donor support should be mobilised. Political willingness and country leadership to align all stakeholders, and long-term commitments by donors for all phases of the plan (preparatory, attack, and consolidation) will be crucial. There will be an important role for non-governmental organisations since these have proven pivotal to national programmes for visceral leishmaniasis and other neglected tropical diseases. To facilitate donor funding, the preparatory phase of the elimination plan should be budgeted. This budget should include the costs for research and development of tools that will enable decentralisation of diagnosis and treatment as well as effective infection prevention. The budget should also include provision for addressing important knowledge gaps in epidemiology, such as updated estimates of visceral leishmaniasis incidence, mortality, the population at risk, and environmental risk maps that can predict outbreaks (table 2). Finally, when tools are developed, knowledge gaps addressed, and regional targets and timelines are set, the attack phase is to be initiated, followed by a consolidation phase to ensure sustainable elimination.

Visceral leishmaniasis should no longer be deprioritised in east Africa. Mobilising stakeholders to support WHO's 2021–30 neglected tropical diseases road map goal for visceral leishmaniasis in east Africa is an important opportunity to re-align health-care priorities and to more effectively address the severe neglect, poverty, and inequity in the region, in accordance with the Sustainable Development Goals.

## Declaration of interests

We declare no competing interests.
