# Supplementary material for: Towards the elimination of visceral leishmaniasis as a public health problem in east Africa: reflections on an enhanced control strategy and a call for action
Source: Lancet Glob Health. 2021 Nov 16;9(12):e1763–9. doi: 10.1016/S2214-109X(21)00392-2 (PMC8609279; doi:10.1016/S2214-109X(21)00392-2)

THE LANCET
Global Health

Supplementary appendix

This appendix formed part of the original submission and has been peer reviewed. We post it as supplied by the authors.

Supplement to: Alvar J, den Boer M, Dagne DA. Towards the elimination of visceral leishmaniasis as a public health problem in east Africa: reflections on an enhanced control strategy and a call for action. *Lancet Glob Health* 2021; **9:** e1763–69.

Table of Contents

Incidence trend by countries and regions, (A) Asia, (B) Africa ……………………………………..page 2

Incidence trend by countries and regions, (A) Asia, (B) Africa

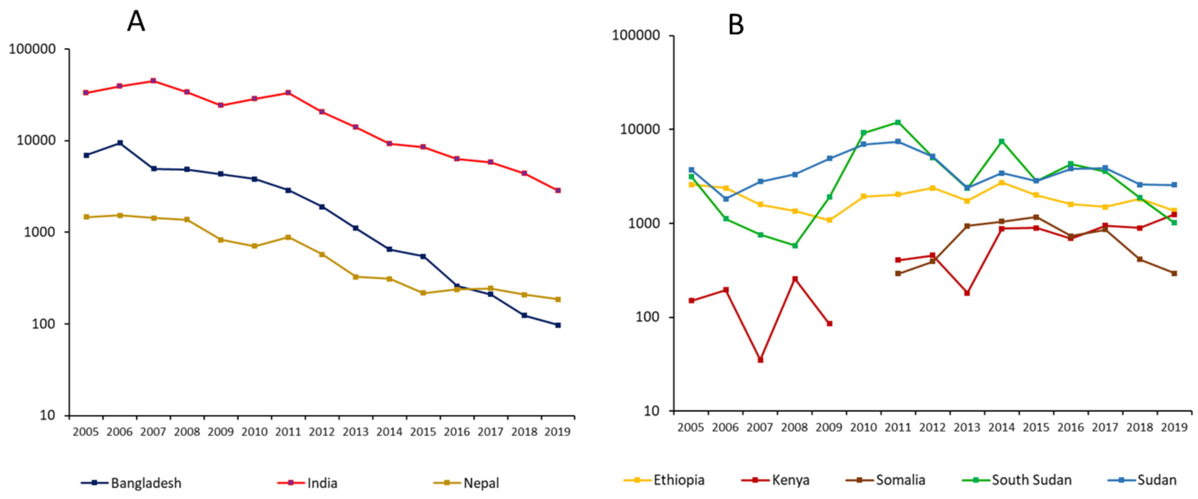

Supplement: Supplementary appendix [file mmc1.pdf]
